# Attainment of low disease activity and remission targets reduces the risk of severe flare and new damage in childhood lupus

**DOI:** 10.1093/rheumatology/keab915

**Published:** 2021-12-11

**Authors:** Eve M D Smith, Kukatharmini Tharmaratnam, Eslam Al-Abadi, Kate Armon, Kathryn Bailey, Mary Brennan, Coziana Ciurtin, Janet Gardner-Medwin, Kirsty E Haslam, Daniel Hawley, Alice Leahy, Valentina Leone, Gulshan Malik, Zoe McLaren, Clarissa Pilkington, Athimalaipet V Ramanan, Satyapal Rangaraj, Annie Ratcliffe, Philip Riley, Ethan Sen, Arani Sridhar, Nick Wilkinson, Christian M Hedrich, Andrea Jorgensen, Michael W Beresford

**Affiliations:** Department of Women's & Children's Health, Institute of Life Course and Medical Sciences, University of Liverpool; Department of Paediatric Rheumatology, Alder Hey Children's NHS Foundation Trust Hospital; Department of Health Data Science, Institute of Population Health, University of Liverpool, Liverpool; Department of Rheumatology, Birmingham Children’s Hospital, Birmingham; Department of Paediatric Rheumatology, Cambridge University Hospitals, Cambridge; Department of Paediatric Rheumatology, Oxford University Hospitals NHS Foundation Trust, Oxford; Department of Paediatric Rheumatology, Royal Hospital for Sick Children, Edinburgh; Department of Rheumatology, Centre for Adolescent Rheumatology, University College London, London; Department of Child Health, University of Glasgow, Glasgow; Department of Paediatrics, Bradford Royal Infirmary, Bradford; Department of Paediatric Rheumatology, Sheffield Children’s Hospital, Sheffield; Department of Paediatric Rheumatology, Southampton General Hospital, Southampton; Department of Paediatric Rheumatology, Leeds Children Hospital, Leeds; Department of Paediatrics, Royal Aberdeen Children’s Hospital, Aberdeen; Rheumatology Department, Aintree University Hospital, Liverpool; Department of Paediatric Rheumatology, Great Ormond Street Hospital, London; Department of Paediatric Rheumatology, University Hospitals Bristol NHS Foundation Trust & Bristol Medical School, University of Bristol, Bristol; Department of Paediatric Rheumatology, Nottingham University Hospitals, Nottingham; Department of Paediatrics, Musgrove Park Hospital, Taunton; Department of Paediatric Rheumatology, Royal Manchester Children’s Hospital, Manchester; Department of Paediatric Rheumatology, Great North Children’s Hospital & Faculty of Medical Sciences, Newcastle University, Newcastle upon Tyne; Department of Paediatrics, Leicester Children’s Hospital, University Hospitals of Leicester NHS trust, Leicester; Department of Paediatric Rheumatology, Guy's & St Thomas's NHS Foundation Trust, Evelina Children's Hospital, London, UK; Department of Women's & Children's Health, Institute of Life Course and Medical Sciences, University of Liverpool; Department of Paediatric Rheumatology, Alder Hey Children's NHS Foundation Trust Hospital; Department of Health Data Science, Institute of Population Health, University of Liverpool, Liverpool; Department of Women's & Children's Health, Institute of Life Course and Medical Sciences, University of Liverpool; Department of Paediatric Rheumatology, Alder Hey Children's NHS Foundation Trust Hospital

**Keywords:** Childhood-SLE, cSLE, treat-to-target, T2T, low disease activity, remission

## Abstract

**Objectives:**

To assess the achievability and effect of attaining low disease activity (LDA) or remission in childhood-onset SLE (cSLE).

**Methods:**

Attainment of three adult-SLE derived definitions of LDA (LLDAS, LA, Toronto-LDA), and four definitions of remission (clinical-SLEDAI-defined remission on/off treatment, pBILAG-defined remission on/off treatment) was assessed in UK JSLE Cohort Study patients longitudinally. Prentice–Williams–Petersen gap recurrent event models assessed the impact of LDA/remission attainment on severe flare/new damage.

**Results:**

LLDAS, LA and Toronto-LDA targets were reached in 67%, 73% and 32% of patients, after a median of 18, 15 or 17 months, respectively. Cumulatively, LLDAS, LA and Toronto-LDA was attained for a median of 23%, 31% and 19% of total follow-up-time, respectively. Remission on-treatment was more common (61% cSLEDAI-defined, 42% pBILAG-defined) than remission off-treatment (31% cSLEDAI-defined, 21% pBILAG-defined). Attainment of all target states, and disease duration (>1 year), significantly reduced the hazard of severe flare (*P* < 0.001). As cumulative time in each target increased, hazard of severe flare progressively reduced. LLDAS attainment reduced the hazard of severe flare more than LA or Toronto-LDA (*P* < 0.001). Attainment of LLDAS and all remission definitions led to a statistically comparable reduction in the hazards of severe flare (*P* > 0.05). Attainment of all targets reduced the hazards of new damage (*P* < 0.05).

**Conclusions:**

This is the first study demonstrating that adult-SLE-derived definitions of LDA/remission are achievable in cSLE, significantly reducing risk of severe flare/new damage. Of the LDA definitions, LLDAS performed best, leading to a statistically comparable reduction in the hazards of severe flare to attainment of clinical remission.

Rheumatology key messages

- Adult-SLE definitions of LDA/remission are achievable in cSLE, significantly reducing the risk of flares/damage.
- In cSLE, long-term target assessment and therapeutic adjustment is required, to minimize severe flare risk.
- Adaptation of existing LDA/remission targets could be considered to improve the applicability to cSLE.

## Introduction

Childhood-onset SLE (cSLE, also known as juvenile-onset SLE or JSLE) is a multisystem chronic autoimmune/autoinflammatory disorder. Children are more severely affected than adults [1–3]. Treatment aims to prevent organ damage and optimize health-related quality of life (HRQOL) through minimizing disease activity, comorbidities and drug toxicity [4]. Persistent disease activity is associated with rapid accrual of organ damage, protracted corticosteroid therapy and increased mortality [5]. A treat-to-target (T2T) approach, where treatment is escalated until a specific target is achieved, and re-escalated if the target is lost, has been proposed as a strategy to improve adult-onset SLE (aSLE) outcomes [6]. However, initiatives focusing on cSLE are lacking.

International principles and recommendations for T2T in aSLE have highlighted the need for validated remission and low disease activity (LDA) definitions, to enable a T2T approach [7]. The Definition Of Remission In SLE (DORIS) international task force has developed consensus-based ‘basic principles’ that disease remission definitions should adhere to [8]. A number of LDA definitions have been proposed, with the Asia Pacific Lupus Consortium producing the most widely accepted lupus low disease activity state (LLDAS) definition, based on the principle of ‘tolerable’ disease activity on stable treatment, with low corticosteroid doses and reduced likelihood of adverse outcomes. LLDAS attainment is associated with reduced SLICC/ACR Damage Index (SDI)-defined organ damage [9–14], fewer flares [15], glucocorticoid sparing [11, 15], improved HRQOL [16] and reduced healthcare costs [17]. Failure to achieve LLDAS within 6 months of diagnosis is associated with early damage [10]. Some studies comparing LDA and remission attainment have demonstrated lower damage accrual, and greater glucocorticoid sparing when remission is achieved [9, 12, 18].

‘Targeting disease, Agreeing Recommendations and reducing Glucocorticoids through Effective Treatment, in LUPUS’ (TARGET LUPUS©) aims to develop a cSLE T2T clinical trial. Currently, no data robustly define appropriate cSLE T2T target(s). The current study aims to assess the achievability of aSLE LDA and remission targets in participants of the UK JSLE Cohort Study [19], investigating the impact of attaining such targets in terms of disease flares and new damage.

## Methods

### Patients

The UK JSLE Cohort Study [19] collects longitudinal data from 22 paediatric rheumatology centres. Patients included fulfilled the following: (i) monitored between 2006 and 2020, (ii) aged ≤18 years at diagnosis, and (iii) fulfilled ≥4 ACR-SLE classification criteria [20]. Written informed patient assent/consent and parental consent was obtained to participate in the UK JSLE Cohort Study, and full ethical approval for the study was in place (National Research Ethics Service North West, Liverpool, UK, reference 06/Q1502/77). Research was carried out in accordance with the Declaration of Helsinki.

### Clinical data for assessing attainment of the targets and outcomes

At the time of recruitment to the study (usually at diagnosis) and during follow-up, the following data items were considered: (i) demographics (gender, ethnicity, diagnosis age and disease duration at each visit), (ii) ACR-SLE classification criteria, (iii) cSLE disease activity [full SLEDAI-2K score (SLEDAI-2K), clinical-SLEDAI-2K score (cSLEDAI) and pBILAG2004 score], (iv) SDI score, and (v) blood/urine laboratory parameters for calculating SLEDAI-2K/pBILAG scores.

### LDA and remission targets

Attainment of three LDA and four remission definitions was assessed at each visit.

LDA definitions were as follows:

- LLDAS: (i) SLEDAI-2K≤4, ‘no major active organ involvement’ (renal, central nervous system, cardiopulmonary, vasculitis, fever), haemolytic anaemia or gastrointestinal involvement; (ii) no new features of lupus activity compared with previous assessment; (iii) physician global assessment ≤1 (0–3 scale); (iv) prednisolone dose ≤7.5 mg/day, no intravenous methylprednisolone; and (v) tolerated standard maintenance immunosuppressive drugs/biological agents, excluding investigational drugs [14].
- LA: as per the LLDAS definition [21, 22] with criterion (i) limited to SLEDAI-2K≤4, and exclusion of criterion (ii).
- Toronto-Low Disease Activity (Toronto-LDA): (i) cSLEDAI-2K score <3 (with or without high dsDNA-antibody levels, or low C3 or C4), only one manifestation of rash, alopecia, mucosal ulcers, pleurisy, pericarditis, fever, thrombocytopenia and leukopoenia; (ii) no corticosteroids; and (iii) no immunomodulators (antimalarials were permitted) [23].

Remission definitions largely followed the DORIS recommendations. The only exception was that in the current study we do not pre-specify the duration of remission target attainment required for the remission targets to be reached. In contrast, the DORIS taskforce recommended that remission in SLE should be a ‘durable state’ [8]. Remission targets were defined as follows:

- Remission on treatment based upon clinical-SLEDAI (remission on-treatment SLEDAI-defined) or pBILAG scores (remission on-treatment BILAG-defined): (i) cSLEDAI = 0 or pBILAG domains scoring D or E; (ii) physician global assessment ≤0.5; (iii) prednisolone dose ≤5 mg/day, no intravenous methylprednisolone; and (iv) tolerated standard maintenance doses of immunosuppressive drugs/biological agents, excluding investigational drugs.
- Remission off-treatment based upon clinical-SLEDAI (remission off-treatment SLEDAI-defined) or pBILAG scores (remission off-treatment BILAG-defined): excluded criteria (iii) and (iv) from the above definitions (antimalarials allowable).

### Outcome variable definitions

Two outcomes were assessed with respect to target attainment: (i) severe flare (BILAG A or B in any organ domain during follow-up); and (ii) new damage (SDI score increase by ≥1 unit).

### Statistical analysis

Descriptive analyses included median values, interquartile ranges (IQR), counts and percentages.

### Predictors of achieving the targets

Univariable logistical regression models assessed whether clinico-demographic factors (listed in Supplementary Box S1, available at *Rheumatology* online) at study recruitment could help to characterize patients that would be more likely to reach the different target definitions for a longer proportion of their follow-up time. The cumulative length of time in each target was calculated for each patient and divided by their total follow-up time, to determine the percentage of cumulative time that each individual patients spent in each target. Within the logistic regression models, patients who reached each target definition for more than the median percentage cumulative time were defined as ‘achieving a high proportion of follow-up in target’, and compared with all other study patients (those not achieving targets, plus those spending less than median percentage cumulative time in target). Multivariable logistic regression models including factors with *P* < 0.05 in univariable analysis were then fitted using the stepBIC selection method, to identify independent predictors of spending > median percentage cumulative time in target. Where a laboratory test value was missing at study recruitment, the subsequent test value was imputed if available within 6 weeks of the initial visit. Multivariable logistic regression models included patients with complete data for each of the exploratory variables, and therefore there were different numbers of patients in each of the regression models.

### Prentice, Williams and Peterson gap model

Each outcome variable (severe flare/new damage) was considered as a sequence of recurrent events, and Prentice, Williams and Peterson (PWP) gap time models [24–26] were fitted to assess risk of recurrent episodes of severe flare or new damage during follow-up. Supplementary Box S1 provides further details on the PWP gap model. For each outcome, a univariable PWP gap model was fitted including each of the factors in turn (factors listed in Supplementary Box S1). Subsequently, for each target in turn, multivariable PWP gap models were then fitted including all covariates found significant univariately (*P* < 0.05) plus a time-varying covariate to represent whether the patient was in target or not (0: not in target; 1: in target). Further models were fitted where the treatment target was represented by a covariate reflecting percentage cumulative duration of time spent in target. Multivariable models included patients with complete data necessary to assess for attainment of each target, and therefore there were different numbers of patients in each PWP gap model. The resulting models were compared based on the hazard ratios (HRs) using a two-sided Student’s *t*-test for dependent samples, using the h.comp2() function in survcomp [27]. This function compares two HRs from their β values and standard errors (e.g. as computed by a Cox model). The two HRs that were compared were computed from the same survival data, and the Bonferroni correction was applied to account for multiple testing.

The ‘Survival’ package was used to fit the PWP gap time models, the ‘glm()’ function was used to fit logistic regression models, and the ‘stepAIC()’ function in the ‘MASS’ package was used for variable selection in logistic regression models [28].

## Results

### Patients

Four hundred and thirty UK JSLE Cohort Study patients were included (83% female), diagnosed with cSLE at 12.8 (IQR: 10.4, 14.6) years fulfilling five [5, 7] SLE ACR criteria. Data were analysed from 4738 visits, representing 10 visits [5, 15] per patient, over 2.0 (0.7, 4.0) years (Table 1).

**Table 1:** Clinical and demographic features

Clinical and demographic feature	Value
Female gender, *n* (%)	359/430 (83)
Ethnicity, *n* (%)	
White British	218/430 (51)
Asian	129/430 (30)
African/Caribbean	72/430 (17)
Age at diagnosis, median (IQR), years	12.8 (10.4, 14.6)
Disease duration, median (IQR), years	2.0 (0.7, 4.0)
Number of visits per patient, median (IQR)	10 (5, 15)
ACR criteria at diagnosis, median (IQR)	5 (5, 7)
ANA positivity at study recruitment, *n* (%)	396 (92)
Anti-dsDNA positivity at study recruitment, *n* (%)	294 (69)
SDI score at study recruitment (individual patients), *n* (%)	
No damage 0	344 (80)
Mild damage (1)	49 (11)
Moderate damage (2)	9 (2)
Severe (≥3)	8 (2)
SDI score during all follow-up visits, *n* (%)	
No damage (SDI = 0)	3150 (71)
Mild (SDI = 1,2)	1067 (24)
Moderate—severe (SDI > 3)	228 (5)

Self-reported ethnicity information was collected in accordance with the UK National Census categorizations. Data of patients who were of mixed race were grouped with those of the associated ethnic minority group. Ethnicity data not available for 11 patients. SDI score at study recruitment not available for 20 patients. IQR: interquartile range; SDI: SLICC Standardized Damage Index.

### Target attainment

LDA was achieved on at least one occasion by 67% of patients using LLDAS, 73% using LA and 32% using Toronto-LDA definitions. Of the 4738 study visits, LLDAS, LA and Toronto-LDA definitions were achieved in 19%, 29% and 8% of all visits, respectively (Table 2). LLDAS, LA and Toronto-LDA targets were reached after a median of 18, 15 and 17 months respectively. The factors contributing to LDA non-attainment are shown in Supplementary Table S1 (available at *Rheumatology* online) on a per visit basis. For example, in patients with a SLEDAI score of ≤4, the LLDAS definition was not attained at 2143 visits: due to the prednisolone dose being >7.5 mg in 827/2143 (39%) of visits; there being new features of lupus activity compared with previous assessment in 739/2143 (35%); major active organ involvement in 536/2143 (25%); changes to immunosuppression in 226/2143 (11%); and a physician global score of >1 in 107/2143 (5%) of visits. For those not attaining LLDAS the median prednisolone dosage was 10 (IQR: 10–17.5) mg, whereas for those attaining LLDAS the median prednisolone dosage was 5 (2.5–5) mg. Similar data are shown exploring the reasons for non-attainment of LA and Toronto-LDA in Supplementary Table S1, available at *Rheumatology* online.

**Table 2:** Achievability of low disease activity state and remission definitions in cSLE patients

Target attainment during follow-up	Number of patients (%) (*n* = 430)	Number of visits (%) (*n* = 4738)	Time to target attainment, median (IQR), months	Percentage of time in target per patient, median (IQR)	Length of time in target, median (IQR), months
Low disease activity					
LLDAS	286 (67)	918 (19)	18.0 (8.5, 30.8)	22.9 (12.8, 36.8)	10.1 (6.0, 20.1)
LA	314 (73)	1368 (29)	14.6 (7.4, 26.8)	31.4 (15.9, 51.5)	13.7 (7.0, 27.6)
Toronto-LDA	136 (32)	393 (8)	17.0 (2.9, 37.7)	18.6 (9.2, 42.5)	9.9 (4.4, 22.1)
Remission definitions					
On-treatment (SLEDAI-defined)	261 (61)	848 (18)	16.8 (8.5, 29.9)	27.9 (14.8, 45.6)	12.1 (6.0, 22.8)
On-treatment (BILAG-defined)	182 (42)	469 (10)	20.7 (11, 38.0)	18.8 (10.3, 33.0)	10.3 (4.8, 18.2)
Off-treatment (SLEDAI-defined)^a^	134 (31)	351 (7)	21.5 (5.5, 39.5)	15.4 (7.7, 40.8)	9.6 (4.5, 20.0)
Off-treatment (BILAG-defined)^a^	90 (21)	200 (4)	24.3 (8.5, 41.8)	14.8 (6.7, 25.5)	8.7 (3.2, 16.1)

a For those achieving remission off-treatment, hydroxychloroquine was still allowable and was prescribed during 37.6% of all visits when in SLEDAI-defined remission off-treatment, and 42.5% of all visits when in BILAG-defined remission off-treatment. BILAG: definition of remission based upon the BILAG score; cSLE: childhood-onset SLE; IQR: interquartile range; LA: low activity; LDA: low disease activity; LLDAS: lupus low disease activity state; SLEDAI: definition of remission based upon the SLEDAI.

Remission on-treatment was easier to achieve (61% SLEDAI-defined, 42% pBILAG-defined) than remission off-treatment (31% SLEDAI-defined, 21% pBILAG-defined). Of 4738 study visits, remission on-treatment was achieved in 18% (SLEDAI-defined) and 10% (pBILAG-defined) of visits. Remission off-treatment was only achieved in 7% (SLEDAI-defined) or 4% (pBILAG-defined) of visits. Remission on-treatment (SLEDAI and BILAG defined) was reached for the first time after a median of 17 and 21 months, respectively, with remission off-treatment (SLEDAI and BILAG defined) attained at 22 and 24 months, respectively (Table 2). At each visit, there was overlap between attainment of the different LDA and remission targets (see Supplementary Fig. S1, available at *Rheumatology* online).

### Predictors of achieving a ‘high proportion of follow-up time in target’

Patients were defined as ‘achieving a high proportion of follow-up in target’ if the cumulative time that they spent in target was more than the median percentage cumulative time in target for the cohort as a whole; 125/430 (29%) spent a high proportion of follow-up time in LLDAS, 142/430 (33%) in LA, 60/430 (14%) in Toronto-LDA, 124/430 (29%) in SLEDAI-defined remission on-treatment, 84/430 (20%) in BILAG-defined remission on-treatment, 59/430 (14%) in SLEDAI-defined remission off-treatment and 39/430 (9%) in BILAG-defined remission off-treatment (Supplementary Table S2, available at *Rheumatology* online). Those not achieving the targets, plus those spending ≤ median percentage cumulative time in target were grouped, and hence the number of patients classified as achieving a ‘high proportion of follow-up in target’ is less than the expected 50% for each target.

Supplementary Table S3 (available at *Rheumatology* online) presents results of univariable analyses. Table 3 includes a summary of factors significantly associated with ‘achieving a high proportion of follow-up’ in each of the LDA definitions in multivariable analysis. Independent predictors of achieving a high proportion of follow-up time in LLDAS included Asian or White British (*vs* African/Caribbean) ethnicity, with low C3 reducing the likelihood of achieving this. A similar pattern was seen for the LA target. However, having an ESR ≤50 mm/h (as compared with ESR >50 mm/h) also increased likelihood of spending greater time in LA target. For Toronto-LDA, only low C3 reduced likelihood of spending a high proportion of time in target (all *P* < 0.05).

**Table 3:** Multivariable logistic-regression models showing predictors (at diagnosis) of spending a high-proportion of follow-up in target

	OR (95% CI)	*P*-value
LLDAS model (*n* = 334)		
Low C3 (<1.04 g/l)	0.45 (0.27, 0.75)	**0.002**
Ethnicity^a^		
Asian	3.70 (1.50, 9.10)	**0.004**
White British	3.02 (1.28, 7.15)	**0.012**
LA model (*n* = 298)		
Low C3 (<1.04 g/l)	0.33 (0.19, 0.58)	**<0.001**
ESR ≤50 mm/h	4.64 (1.42, 15.18)	**0.011**
Ethnicity^a^		
Asian	3.53 (1.37, 9.10)	**0.009**
White British	3.64 (1.50, 8.83)	**0.004**
Toronto-LDA model (*n* = 341)		
Low C3 (<1.04 g/l)	0.30 (0.15, 0.60)	**0.001**
Remission on-treatment model (SLEDAI-defined, *n* = 303)		
Low C3 (<1.04 g/l)	0.44 (0.25, 0.76)	**0.004**
ESR ≤50 mm/h	7.08 (1.84, 27.30)	**0.004**
Remission on-treatment model (BILAG-defined, *n* = 334)		
Low C3 (<1.04 g/l)	0.40 (0.23, 0.70)	**0.001**
Ethnicity^a^		
Asian	5.20 (1.70, 15.84)	**0.004**
White British	3.09 (1.04, 9.21)	**0.043**
Remission off-treatment model (SLEDAI-defined, *n* = 341)		
Low C3 (<1.04 g/l)	0.51 (0.26, 0.99)	**0.049**
BILAG-defined renal involvement	0.32 (0.13, 0.80)	**0.014**
Remission off-treatment model (BILAG-defined, *n* = 392)		
Lymphopenia^b^	0.46 (0.22, 0.97)	**0.041**

Different patient numbers in each regression model, only patients with complete data included.

a African/Caribbean ethnicity is the reference variable.

b Lymphopenia is <1.5  × 10^9^/l. Variables selected using stepBIC selection method (including variables with *P* < 0.05 univariately). C4 excluded as highly correlated with C3. Total numerical BILAG score excluded as highly correlated with individual BILAG organ domains. Total SLEDAI score excluded as highly correlated with the outcome measures. LA: low activity; LDA: low disease activity; LLDAS: lupus low disease activity state; OR: odds ratio. Significant p-values (p<0.05) are shown in bold text.

Table 3 also presents multivariable analysis data for each remission definition, demonstrating that low C3 reduced and having an ESR of ≤50 mm/h increased the likelihood that a patient would spend a high proportion of time in SLEDAI-defined remission on-treatment. For pBILAG-defined remission on-treatment, being of Asian or White British ethnicity (*vs* African/Caribbean) increased, and low C3 reduced the likelihood of spending a high proportion of time in target. Both low C3 and BILAG-defined renal involvement made it less likely that a patient will spend a high proportion of time in SLEDAI-defined remission off-treatment. The likelihood of achieving BILAG-defined remission off-treatment was reduced by presence of lymphopenia (all *P* < 0.05). These analyses help to characterize patients that are more likely to reach the different target definitions for a longer proportion of their follow-up time.

### Effect of achieving the targets on hazards of ‘severe flare’

#### Univariable analysis

Table 4 presents HRs, 95% CI and *P*-values for univariable analyses of factors associated with severe flare risk. Severe flare was present in 2013/4738 visits (42.5%). The following factors reduced the hazards of severe flare: duration of disease >1 year; being of Asian or White British ethnicity (*vs* African/Caribbean); attainment of each LDA target; attainment of SLEDAI-defined remission on/off-treatment; and spending a greater proportion of cumulative time in each LDA/remission target state. In contrast, the following factors increased the hazards of severe flare: SDI scores of ≥1 at the time of study recruitment; and increasing SDI scores during follow-up.

**Table 4:** Univariable PWP gap models assessing impact of demographic factors/target attainment on ‘severe flare’ and new damage

	Severe flare		New damage	
	HR (95% CI)	*P*-value	HR (95% CI)	*P*-value
Sex (female)	0.99 (0.80, 1.21)	0.895	1.18 (0.70, 2.00)	0.532
Disease duration (>1 year)	0.80 (0.74, 0.86)	**<0.001**	0.95 (0.83, 1.09)	0.456
Ethnicity^a^				
Asian	0.78 (0.63, 0.98)	**0.031**	0.92 (0.54, 1.58)	0.760
White British	0.79 (0.64, 0.97)	**0.024**	0.75 (0.44, 1.26)	0.270
SDI score of ≥1 at study recruitment	1.13 (1.02, 1.25)	**0.015**	NA2	NA2
Increasing SDI score during follow-up	1.10 (1.04, 1.17)	**<0.001**	NA2	NA2
Target state attainment at any time point^b^:				
LLDAS	0.14 (0.11, 0.19)	**<0.001**	0.24 (0.12, 0.48)	**<0.001**
LA	0.31 (0.26, 0.37)	**<0.001**	0.44 (0.29, 0.67)	**<0.001**
Toronto-LDA	0.17 (0.12, 0.25)	**<0.001**	0.35 (0.15, 0.83)	**0.017**
Remission on-treatment (SLEDAI-defined)	0.17 (0.13, 0.22)	**<0.001**	0.27 (0.14, 0.50)	**<0.001**
Remission off-treatment (BILAG-defined)	NA1	NA1	0.10 (0.03, 0.42)	**0.001**
Remission off-treatment (SLEDAI-defined)	0.10 (0.07, 0.16)	**<0.001**	0.33 (0.28, 0.40)	**<0.001**
Remission off-treatment (BILAG-defined)	NA1	NA1	NA3	NA3
Percentage of the cumulative duration of follow-up in each target state^b^^,^^c^			NA4	
LLDAS	0.962 (0.952,0.973)	**<0.001**		
LA	0.973 (0.967,0.980)	**<0.001**		
Toronto-LDA	0.975 (0.965,0.985)	**<0.001**		
Remission on-treatment (SLEDAI-defined)	0.969 (0.961,0.976)	**<0.001**		
Remission on-treatment (BILAG-defined)	0.951 (0.939,0.963)	**<0.001**		
Remission off-treatment (SLEDAI-defined)	0.974 (0.963,0.984)	**<0.001**		
Remission off-treatment (BILAG-defined)	0.945 (0.927,0.964)	**<0.001**		

NA1: modelling not possible as pBILAG used to define flare. NA2: modelling not possible as SDI-score used to define damage. NA3: modelling not possible, as no patients who achieved BILAG-defined remission developed new damage. NA4: modelling not possible, small number of patients accruing new damage while in target. Cl: confidence interval; HR: hazards ratio; LA: low activity; LDA: low disease activity; LLDAS: lupus low disease activity state; PWP: Prentice–Williams–Peterson; SDI: SLICC Standardized Damage Index. Significant p-values (p<0.05) are shown in bold text.

a African/Caribbean ethnicity is the reference variable.

b Target state attainment, and cumulative duration of follow-up in target are time varying covariates.

c Models assessed the percentage of the cumulative duration of follow-up in each target state. The HR’s shown are for each 1% cumulative duration of follow-up in each target state.

To aid interpretation of the effect of spending increasing periods of time in target, Table 5 summarizes the HR for severe flare for various levels of cumulative percentage time in target. For example, increasing the cumulative duration of time in LLDAS target from 10% to 80% of follow-up time reduces the hazards of severe flare from 0.68 down to 0.05 (Table 5).

**Table 5:** Hazard ratios for risk of ‘severe flare’ with respect to increasing percentage time in target

	Increasing cumulative duration of time in target and hazards ratios for ‘severe flare’					
	10%	20%	40%	50%	60%	80%
LLDAS	0.68	0.46	0.21	0.14	0.10	0.05
LA	0.76	0.58	0.34	0.26	0.20	0.11
Toronto-LDA	0.78	0.60	0.36	0.28	0.22	0.13
Remission on-Treatment (SLEDAI-defined)	0.73	0.53	0.28	0.20	0.15	0.08
Remission on-Treatment (BILAG-defined)	0.60	0.36	0.13	0.08	0.05	0.02
Remission off-Treatment (SLEDAI-defined)	0.77	0.59	0.34	0.26	0.20	0.12
Remission off-Treatment (BILAG-defined)	0.57	0.32	0.11	0.06	0.03	0.01

All values are hazard ratios. LA: low activity; LDA: low disease activity; LLDAS: lupus low disease activity state.

#### Multivariable analysis

Multivariable models explored ‘target attainment at any time point’ or ‘percentage of cumulative follow-up in target’ (Table 6), and whether this impacted upon the hazards of severe flare during follow-up. Clinico-demographic factors significant in the univariate analysis (Table 4, factors with *P* < 0.05 univariately) were included. The co-variates ‘target attainment at any time point’ and ‘percentage of cumulative follow-up in target’ are derived from the same information. Therefore, models considering these co-variates were fitted separately.

**Table 6:** Multivariable PWP gap models for ‘severe flare’

	LLDAS (*n* = 286)		LA (*n* = 314)		Toronto-LDA (*n* = 136)		Remission on-treatment (SLEDAI-defined) (*n* = 261)		Remission off-treatment (SLEDAI-defined) (*n* = 134)		Remission on-treatment (BILAG-defined) (*n* = 182)		Remission off-treatment (BILAG-defined) (*n* = 90)	
	HR (95% CI)	*P*-value	HR (95% CI)	*P*-value	HR (95% CI)	*P*-value	HR (95% CI)	*P*-value	HR (95% CI)	*P*-value	HR (95% CI)	*P*-value	HR (95% CI)	*P*-value
Multivariate models including ‘target attainment at any time point’^a^														

	Model 1		Model 2		Model 3		Model 4		Model 5					

Disease duration (>1 year)	**0.82 (0.77, 0.88)**	**<0.001**	**0.82 (0.77, 0.88)**	**<0.001**	**0.83 (0.77, 0.89)**	**<0.001**	**0.83 (0.77, 0.88)**	**<0.001**	**0.83 (0.77, 0.90)**	**<0.001**	NA		NA	
Ethnicity^b^														
Asian	0.85 (0.70, 1.02)	0.081	0.85 (0.71, 1.03)	0.089	**0.78 (0.63, 0.95)**	**0.014**	0.84 (0.70, 1.02)	0.071	**0.78 (0.63, 0.95)**	**0.016**				
White British	0.86 (0.72, 1.02)	0.081	0.86 (0.72, 1.02)	0.084	**0.78 (0.65, 0.94)**	**0.001**	0.84 (0.70, 1.00)	0.050	**0.79 (0.65, 0.95)**	**0.013**				
Target state attainment at any time point	**0.15 (0.11, 0.20)**	**<0.001**	**0.33 (0.28, 0.39)**	**<0.001**	**0.21 (0.15, 0.31)**	**<0.001**	**0.19 (0.15, 0.24)**	**<0.001**	**0.13 (0.09, 0.20)**	**<0.001**				
Increasing SDI score during f/u	**1.10 (1.05, 1.14)**	**<0.001**	**1.10 (1.06, 1.14)**	**<0.001**	**1.10 (1.06, 1.14)**	**<0.001**	**1.10 (1.06, 1.14)**	**<0.001**	**1.09 (1.04, 1.15)**	**<0.001**				

Multivariable PWP gap models with ‘percentage of the cumulative duration of follow-up’ in each target state^c^														

	Model 6		Model 7		Model 8		Model 9		Model 10		Model 11		Model 12	

Disease duration (>1 year)	**0.83 (0.78, 0.90)**	**<0.001**	**0.84 (0.78, 0.90)**	**<0.001**	**0.82 (0.76, 0.89)**	**<0.001**	**0.84 (0.79, 0.91)**	**<0.001**	**0.84 (0.78, 0.90)**	**<0.001**	**0.82 (0.75, 0.88)**	**<0.001**	**0.82 (0.76, 0.89)**	**<0.001**
Ethnicity^b^														
Asian	0.85 (0.70, 1.03)	0.095	0.84 (0.69, 1.02)	0.084	**0.76 (0.62, 0.92)**	**0.006**	0.83 (0.68, 1.00)	0.051	0.83 (0.69, 1.01)	0.059	**0.76 (0.62, 0.92)**	**0.006**	**0.77 (0.63, 0.95)**	**0.013**
White British	0.85 (0.71, 1.01)	0.072	0.84 (0.70, 1.00)	0.056	**0.77 (0.64, 0.93)**	**0.006**	**0.80 (0.67, 0.96)**	**0.016**	**0.82 (0.69, 0.97)**	**0.022**	**0.77 (0.64, 0.93)**	**0.006**	**0.79 (0.66, 0.95)**	**0.012**
Percentage cumulative duration in each target^c,d^	**0.97 (0.96, 0.98)**	**<0.001**	**0.98 (0.97, 0.99)**	**<0.001**	0.99 (0.98, 1.0)	0.069	**0.98 (0.97, 0.99)**	**<0.001**	**0.96 (0.95, 0.97)**	**<0.001**	0.99 (0.98, 1.0)	0.124	**0.97 (0.95, 0.98)**	**<0.001**
Increasing SDI score during f/u^d^	**1.09 (1.01, 1.14)**	**<0.001**	**1.09 (1.05, 1.14)**	**<0.001**	**1.10 (1.05, 1.16)**	**<0.001**	**1.09 (1.05, 1.14)**	**<0.001**	**1.10 (1.05, 1.14)**	**<0.001**	**1.11 (1.05, 1.16)**	**<0.001**	**1.10 (1.05, 1.16)**	**<0.001**

a Within these models, target achieved at least once.

b African/Caribbean ethnicity is the reference variable.

c Percentage cumulative duration in target is relative to total follow-up period. HR’s relate to each 1% increase of cumulative time in target. Those with complete data needed for target assessment included, leading to different numbers per PWP-Gap model. ^d^Time-varying covariates: percentage cumulative duration in target, increasing SDI-score during follow-up. BILAG: British Isles Lupus assessment group; Cl: confidence interval; f/u: follow-up; HR: hazards ratio; LA: low activity; LDA: low disease activity; LLDAS: lupus low disease activity state; NA: models could not be fitted; PWP: Prentice–Williams–Peterson. BILAG-score used to define severe flare in all models. The HR, 95% CI and p-value are shown in bold text in instances where a co-variate is significant within the multivariate models.

The upper section of Table 6 summarizes that for all seven LDA and remission targets ‘target attainment at any time point’, and having a disease duration of >1 year, significantly reduced the hazard of severe flare during follow-up in all models. In the Toronto-LDA and SLEDAI-defined remission off-treatment models, being of Asian or White British ethnicity (*vs* African/Caribbean ethnicity) independently reduced the hazard of severe flare (*P* < 0.05). For all models, increasing SDI score increased the hazards of severe flare (*P* < 0.001) during follow-up.

The lower section of Table 6 demonstrates that the ‘percentage of the cumulative duration of follow-up’ in LLDAS, LA, SLEDAI-defined remission on-treatment, and SLEDAI-defined remission off-treatment and BILAG-defined remission off-treatment all independently reduced the hazard of severe flare (*P* < 0.001). Having a disease duration of >1 year reduced the hazard of severe flare in all models (*P* < 0.001), but the impact of ethnicity varied between models. Again, increasing SDI score during follow-up increased the hazards of severe flare in all models (*P* < 0.001).

#### Comparison of LDA or remission attainment and ‘severe flare’

The HRs for ‘target attainment at any time point’ (Table 6) or ‘percentage of the cumulative duration of follow-up in target’ (Table 6) were similar across all target definitions, and therefore the HRs were compared statistically to see if a difference could be detected. The hazard of severe flare was lower when LLDAS was achieved, as opposed to LA (*P*_c_ < 0.001, Supplementary Table S4, available at *Rheumatology* online), highlighting that achievement of LLDAS is more protective against severe flare. There was no significant difference between the hazards of severe flare when attainment of LLDAS and all definitions of clinical remission (SLEDAI or BILAG defined, on/off-treatment) were compared (all *P*_c_ > 0.05), suggesting comparability between attainment of LLDAS and clinical remission definitions as regards the hazards of severe flare. Similar comparisons were undertaken for the HR relating to ‘percentage of the cumulative duration of follow-up’ in different target states (shown in Supplementary Table S5, available at *Rheumatology* online).

### Impact of achieving the targets and new damage

Table 4 presents univariable analyses of variables reducing the risk of new damage. Attainment of each LDA/remission definition significantly reduced the hazards of new damage. Demographic factors were not associated with damage accrual. Therefore, multivariable analysis was not warranted. Comparing the HRs across the different target definitions, there was only a significant difference in HRs for pBILAG-defined remission on-treatment *vs* off-treatment (*P*_c_ < 0.001; Supplementary Table S6, available at *Rheumatology* online), indicating that all other targets do not differ significantly in terms of their effect on new damage. Analysis looking at impact of cumulative duration of remission on ‘new damage’ was not appropriate, due to the low cumulative period.

## Discussion

T2T approaches have been introduced in many conditions, resulting in improved outcomes [29]. The development and validation of targets has been a key enabler for T2T trials. This is the first study to investigate the use of aSLE-derived definitions of LDA and remission in cSLE. We have demonstrated that aSLE targets are achievable in cSLE, reducing the hazards of severe flares and new damage. Balancing attainment and impact on severe flare/damage, the LLDAS definition performed best, demonstrating a statistically equivalent reduction in the hazards of severe flare as compared with attainment of clinical remission targets. On-going longitudinal monitoring of targets is needed, with sustained attainment of the targets demonstrating further reduction in hazards of severe flare/new damage. Future discussion is required between cSLE experts, patients and parents, informed by data such as those presented within this manuscript, to determine whether aSLE targets require any paediatric specific adaptations.

Sixty-seven per cent of patients achieved LLDAS, whereas in the original derivation/validation study [14], LLDAS was achieved by 88.5% of patients. Subsequent validation studies have shown LLDAS to be achieved by ∼75% of patients [30, 31], lasting for ∼50% follow-up on average [9]. LA target was achieved by 73% of UK JSLE patients, for a median of 29% of follow-up time. In two aSLE studies, LA was achieved during 10% of all follow-up intervals [22], with 14.9% of patients in LA target at last follow-up [21]. Toronto-LDA was achieved by 32% of UK JSLE Cohort patients, whereas in the original derivation/validation study, this was achieved by only 12.9% [23]. Overall achievability of LDA targets within UK JSLE Cohort patients appears comparable to the original studies.

61% of UK JSLE Cohort patients achieved SLEDAI-defined remission on-treatment during follow-up, with 31% achieving SLEDAI-defined remission off-treatment. Overall, these definitions were only met for 18% and 7% of the total visits (on/off-treatment respectively). Within aSLE studies, 39–61% achieved SLEDAI-defined remission on-treatment [32, 33], for 10–38% of follow-up visits [22, 32, 33]. Attainment of SLEDAI-defined remission off-treatment was demonstrated in 18–24% of aSLE patients [32, 33], sustained for 2–13% of visits [9, 22]. In the current study, in keeping with aSLE studies, increasing the cumulative time in all LDA and remission target definitions reduced the risk of severe flare [32].

Having a disease duration of >1 year reduced the hazards of severe flare in all multivariable models, highlighting that the first year after diagnosis is a particularly high-risk period. Three disease courses have been described in cSLE: chronic active, relapse remitting and long quiescent, with aggressive treatment in the first 6 months associated with a subsequent long quiescent course [34]. Adult-SLE studies have identified patients with early disease-onset (≤25 years) [35, 36] to be at increased risk of flares. Together these observations support the need for early aggressive management, particularly for patients with early onset disease.

This study demonstrates that attainment of all definitions of LDA and remission reduces the hazards of new damage, in keeping with aSLE studies [9, 12, 13, 31, 32, 37, 38]. Comparing the different LDA targets assessed, reaching LLDAS was more protective against severe flare than LA (*P*_c_ < 0.001), highlighting that domain 2 of the LLDAS definition, namely that there should be ‘no new features of lupus activity compared with previous assessment’, contributes significantly to the protective effect of attaining LLDAS. Secondly, there was no statistically significant difference between the hazards of severe flare when attainment of LLDAS and all definitions of clinical remission were compared (all *P*_c_ > 0.05), suggesting comparability of the effect of LLDAS and clinical remission attainment for risk of severe flare. Disease activity, flare and damage are closely related, with cumulative duration of active disease a known predictor of damage [39–42]. cSLE patients also accrue damage at a faster rate than aSLE patients [39, 41–46]. Institution of T2T approaches, specifically aimed at interrupting this detrimental series of interconnected events, warrants assessment.

Most of the items included in the existing target definitions [8, 14, 21–23] are relevant to cSLE and aSLE. Inclusion of weight-based prednisolone dosage should be considered, informed by analyses specifically comparing the existing LLDAS and LA allowable prednisolone dosage (7.5 mg/daily) with a weight-based alternative. Use of common target definitions across cSLE and aSLE T2T studies could facilitate life course studies, with greater patient numbers. Existing targets [8, 14, 21–23] do not include patient reported outcome measures (PROMS) despite the aSLE T2T international taskforce recommending ‘treatment should aim at ensuring long-term survival, preventing organ damage, and optimizing HRQOL’ and ‘factors negatively influencing HRQOL, such as fatigue, pain and depression, should be addressed’ [6]. Inclusion of PROMs, considering HRQOL, fatigue and drug toxicity, should also therefore be considered when designing a cSLE T2T study.

A qualitative study has recently been undertaken as part of the TARGET LUPUS**^©^**research programme, considering patient/parental views on T2T [47]. Participants differed in how they defined LDA, expressing a preference for being asymptomatic rather than LDA. Most families reported fatigue as a key challenge and were enthusiastic about inclusion of a fatigue PROM. The majority of families suggested targeting of corticosteroid dosage [47]. A recent commentary discussing patient perspectives on T2T suggests that patients are supportive of T2T, but that a holistic approach is necessary, targeting HRQOL, fatigue and drug side effects, in addition to disease activity [48]. In aSLE, attainment of LDA and remission significantly improved HRQOL [37, 49].

Limitations to this study must be acknowledged. The DORIS taskforce recommended that remission should be a ‘durable state’ [8]. We did not pre-specify the duration necessary for remission target attainment in this study, as aSLE cohorts have previously shown ‘durable remission’ to be rare [33], and that even short periods of remission are associated with a reduction in damage [9]. By pre-specifying the duration of remission necessary for target achievement, we would not have been able to assess the effect of transient remission attainment on the hazards of severe flare and damage. Lastly, we assessed clinical remission rather than complete remission, as children who are well or off-treatment do not tend to have blood tests. It is clear from the Venn diagram in Supplementary Fig. S1, available at *Rheumatology* online, that there are high margins of overlap between LDA and clinical remission target definitions. In the future it would be useful to also look at complete remission target attainment as part of a prospective study, to see whether attainment of complete remission would have a much greater effect on risk of severe flare/new damage, and whether there would be less overlap in attainment of complete remission compared with LDA/clinical remission definitions. Our data are collected alongside routine clinical practice, and therefore imputation was used for some missing data points. Due to variation in follow-up time between patients, PWP gap models were employed for longitudinal analyses.

## Conclusions

This study has shown for the first time that aSLE definitions of LDA and remission are achievable in cSLE, and that their attainment reduces the hazards of severe flares and new damage. On-going monitoring of targets during follow-up is important, with sustained target attainment further reducing the hazards of severe flare and new damage. Results from the current study will help to inform future development of a T2T approach for cSLE. They should be considered by cSLE experts, alongside the results of the recent TARGET LUPUS^**©**^ qualitative study [47], which provides insight into patient/parental views on T2T.

## Supplementary Material

keab915_Supplementary_Data

## Data Availability

The data underlying this article will be shared on reasonable request to the corresponding author.
