# Supplementary material for: Attainment of low disease activity and remission targets reduces the risk of severe flare and new damage in childhood lupus
Source: Rheumatology (Oxford). 2021 Dec 11;61(8):3378–89. doi: 10.1093/rheumatology/keab915 (PMC9348762; doi:10.1093/rheumatology/keab915)
Supplement: keab915_Supplementary_Data [file keab915_supplementary_data.docx]

**Supplementary Table S1 - Summary statistics showing the factors contributing to LDA non-attainment on a per visit basis, despite having a SLEDAI score of ≤4 (for LLDAS and LDA targets) or <3 (for Toronto LDA target)**

| **Target** | **Factors contributing to target non-attainment**  **(which are part of the LDA definitions)** | **Visits where target not attained due to the specified factors (n, %)** |
| --- | --- | --- |
| **LLDAS**  (not attained at 2143 visits,  despite SLEDAI score of ≤4) | Prednisolone >7.5 mg day* | 827/2143 (39%) |
|  | New features of lupus activity compared with previous assessment | 739/2143 (35%) |
|  | Major active organ involvement (renal, central nervous system, cardiopulmonary, vasculitis, fever), haemolytic anaemia or gastrointestinal involvement | 536/2143 (25%) |
|  | Changes to immunosuppression (not on maintenance immunosuppression) | 226/2143 (11%) |
|  | Physician global score >1 | 107/2143 (5%) |
| **LA**  (not attained at 1693 visits,  despite SLEDAI score of ≤4) | Prednisone >7.5 mg day** | 827/1693 (49%) |
|  | Changes to immunosuppression (not on maintenance immunosuppression) | 226/1693 (13%) |
|  | Physician global score >1 | 107/1693 (6%) |
| **Toronto-LDA**  (not attained at 1623 visits,  despite SLEDAI score of <3) | On immunosuppression | 1518/1623 (94%) |
|  | On prednisolone | 1057/1623 (65%) |

*For those not attaining LLDAS the median prednisolone dose was 10mg (interquartile range, IQR 10-17.5mg), whereas for those attaining LLDAS the median prednisolone dose was 5mg (interquartile range, IQR 2.5-5mg). ** For those not attaining LDA the median prednisolone dose was 10mg (interquartile range, IQR 10-17.5mg), whereas for those attaining LDA the median prednisolone dose was 5mg (interquartile range, IQR 3-6mg). LLDAS – Lupus low disease activity state. LA - Low Activity. LDA – low disease activity.

**Supplementary Table S2 - Achievability of low disease activity state and remission definitions for a ‘high proportion of follow-up time’ in cSLE patients**

| **Target attainment during follow-up** | **Patients achieving a high proportion of follow-up time in target*** | **Median percentage cumulative time in target**  **(median %, IQR %)** |
| --- | --- | --- |
| **Low disease activity**   - **LLDAS** - **LA** - **Toronto-LDA**   **Remission definitions**   - **On-Treatment model (SLEDAI-defined)** - **On-Treatment model (BILAG-defined)** - **Off-Treatment model (SLEDAI-defined)** - **Off-Treatment model (BILAG-defined)** | 125/430 (29%)  142/430 (33%)  60/430 (14%)  124/430 (29%)  84/430 (20%)  59/430 (14%)  39/430 (9%) | 22.9% [12.8,36.8]  31.4% [15.9,51.5]  18.60% [9.2,42.5]  27.9% [14.8,45.6]  18.8% [10.3,33.0]  15.36% [7.7,40.8]  14.77% [6.7,25.5] |

The overall cumulative length of time spent in each target, as a percentage of the total follow-up time was determined for each patient, with those spending more than the median percentage cumulative time in target defined as patients ‘achieving a high proportion of follow-up time in target’. Those not achieving targets, plus those spending less than median percentage cumulative time in target were grouped together, hence the number of patients classified as achieving a ‘high proportion of follow-up time in target’ for each target are less than the expected 50%. CI -confidence interval. LLDAS – Lupus low disease activity state. LA - Low Activity. LDA – low disease activity. SLEDAI – definition of remission based upon the Systemic Lupus Erythematosus disease activity index. BILAG – definition of remission based upon the British Isles Lupus assessment group score.

**Supplementary Table S3 – Univariable logistic regression models showing clinical and demographic features at the time of study recruitment which are associated with spending > median percentage cumulative time in target**

|  | **LLDAS** | | **LA** | | **Toronto-LDA** | | **Remission ON-Tx model**  **(SLEDAI-defined)** | | **Remission ON-Tx model**  **(BILAG-defined)** | | **Remission OFF-Tx model**  **(SLEDAI-defined)** | | **Remission OFF-Tx model**  **(BILAG-defined)** | |
| --- | --- | --- | --- | --- | --- | --- | --- | --- | --- | --- | --- | --- | --- | --- |
|  | **OR**  **(95% CI)** | **P-value** | **OR**  **(95% CI)** | **P-value** | **OR**  **(95% CI)** | **P-value** | **OR**  **(95% CI)** | **P-value** | **OR**  **(95% CI)** | **P-value** | **OR**  **(95% CI)** | **P-value** | **OR**  **(95% CI)** | **P-value** |
| **Low**  **C3**  **(<1.04 g/l)** | **0.45**  **(0.28, 0.75)** | **0.002** | **0.36**  **(0.22, 0.59)** | **<0.001** | **0.30**  **(0.15, 0.60)** | **0.001** | **0.48**  **(0.29, 0.80)** | **0.005** | **0.40**  **(0.23, 0.70)** | **0.001** | **0.43**  **(0.22, 0.83)** | **0.012** | **0.41**  **(0.19, 0.88)** | **0.022** |
| **Low**  **C4**  **(<0.18 g/l)** | **0.58**  **(0.34, 0.98)** | **0.041** | **0.41**  **(0.24, 0.67)** | **0.001** | **0.26**  **(0.13, 0.52)** | **<0.001** | 0.60  (0.35, 1.03) | 0.062 | 0.56  (0.31, 1.00) | 0.051 | **0.37**  **(0.18, 0.73)** | **0.005** | **0.40**  **(0.18, 0.88)** | **0.023** |
| **dsDNA**  **positive (>20)** | **0.34**  **(0.20, 0.58)** | **<0.001** | **0.46**  **(0.28, 0.77)** | **0.003** | **0.33**  **(0.15, 0.72)** | **0.005** | 0.80  (0.46, 1.38) | 0.420 | 0.72  (0.40, 1.31) | 0.279 | 0.48  (0.22, 1.04) | 0.062 | 0.57  (0.24, 1.35) | 0.199 |
| **Low Platelets**  **(<100**  **x10⁹/l)** | 0.98  (0.45, 2.13) | 0.967 | 1.05  (0.50, 2.19) | 0.894 | 0.90  (0.30, 2.66) | 0.844 | 1.56  (0.75, 3.24) | 0.230 | 1.03  (0.43, 2.46) | 0.947 | 0.92  (0.31, 2.73) | 0.879 | 0.63  (0.14, 2.73) | 0.532 |
| **Leuco-paenia**  **(<4 x10⁹/l)** | 0.97  (0.58, 1.60) | 0.891 | 0.77  (0.47, 1.26) | 0.298 | 0.69  (0.33, 1.43) | 0.320 | 0.84  (0.51, 1.41) | 0.517 | **0.43**  **(0.22, 0.86)** | **0.017** | 0.73  (0.35, 1.52) | 0.401 | **0.27**  **(0.08, 0.91)** | **0.034** |
| **Low Hb (<9 g/dl)** | 0.74  (0.39, 1.42) | 0.368 | 0.66  (0.35, 1.23) | 0.186 | 0.74  (0.30, 1.81) | 0.503 | 0.94  (0.51, 1.75) | 0.848 | 0.95  (0.47, 1.92) | 0.886 | 0.46  (0.16, 1.33) | 0.152 | 0.75  (0.25, 2.19) | 0.593 |
| **Lympho-paenia**  **(<1.5**  **x10⁹/l)** | 0.89  (0.57, 1.38) | 0.598 | 0.86  (0.57, 1.31) | 0.481 | 0.57  (0.31, 1.04) | 0.068 | 0.87  (0.56, 1.35) | 0.542 | 0.75  (0.46, 1.24) | 0.259 | 0.75  (0.41, 1.39) | 0.366 | **0.46**  **(0.22, 0.97)** | **0.041** |
| **ESR**  **≤50mm/hr**  **>50mm/hr** | 1.90  (0.73, 4.37)  1.14  (0.46, 2.78) | 0.206  0.781 | **3.71**  **(1.33,10.4)**  1.70  (0.67, 4.35) | **0.013**  0.265 | 0.72  (0.21, 2.45)  0.58 (0.20, 1.64) | 0.604  0.304 | **3.25**  **(1.16, 9.11)**  1.28  (0.50, 3.28) | **0.025**  0.610 | 1.71  (0.56, 5.23)  1.03  (0.37, 2.85) | 0.349  0.952 | 0.94  (0.26, 3.43)  0.75  (0.24, 2.33) | 0.930  0.624 | 0.52  (0.15, 1.88)  0.36  (0.12, 1.05) | 0.322  0.061 |
| **BILAG defined organ domain involvement** | | | | | | | | | | | | | | |
| **Const-itutional** | 1.01  (0.64, 1.59) | 0.961 | 1.11  (0.72, 1.71) | 0.644 | **0.47**  **(0.24, 0.94)** | **0.034** | 0.97  (0.62, 1.54) | 0.910 | 0.97  (0.58, 1.64) | 0.917 | 0.55  (0.28, 1.07) | 0.078 | 0.78  (0.37, 1.65) | 0.514 |
| **Muco-cutaneous** | 1.00  (0.65, 1.53) | 0.984 | 0.93  (0.62, 1.42) | 0.751 | 0.61  (0.34, 1.12) | 0.110 | 1.01  (0.66, 1.56) | 0.950 | 1.16  (0.71, 1.89) | 0.555 | 0.76  (0.42, 1.36) | 0.352 | 0.81  (0.40, 1.63) | 0.558 |
| **Neuro-psychiatric** | 0.97  (0.48, 1.97) | 0.940 | 1.14  (0.59, 2.22) | 0.696 | 0.45  (0.13, 1.49) | 0.191 | 0.99  (0.49, 1.99) | 0.968 | 0.97  (0.43, 2.17) | 0.933 | 0.84  (0.31, 2.22) | 0.719 | 1.41  (0.52, 3.82) | 0.502 |
| **Musculo-skeletal** | 0.72  (0.45, 1.15) | 0.169 | 0.82  (0.52, 1.28) | 0.376 | 0.69  (0.37, 1.31) | 0.258 | 0.73  (0.45, 1.17) | 0.190 | 0.64  (0.37, 1.13) | 0.123 | 0.79  (0.42, 1.48) | 0.457 | 0.49  (0.21, 1.15) | 0.102 |
| **Cardio-respiratory** | 0.84  (0.43, 1.64) | 0.611 | 0.77  (0.40, 1.47) | 0.423 | **0.11**  **(0.02, 0.82)** | **0.031** | 0.85  (0.44, 1.66) | 0.638 | 0.89  (0.42, 1.92) | 0.771 | 0.24  (0.06, 1.00) | 0.050 | 0.39  (0.09, 1.65) | 0.200 |
| **Gastro-intestinal** | 1.13  (0.42, 3.05) | 0.806 | 1.50  (0.59, 3.82) | 0.392 | 1.16  (0.33, 4.12) | 0.813 | 1.15  (0.43, 3.09) | 0.787 | 0.76  (0.22, 2.68) | 0.674 | 1.19  (0.34, 4.21) | 0.789 | 1.95  (0.54, 7.02) | 0.305 |
| **Ophthal-mic** | 7.48  (0.77, 72.6) | 0.083 | 6.19  (0.64, 60.1) | 0.116 | 2.07  (0.21, 20.3) | 0.531 | 7.56  (0.78, 73.4) | 0.081 | 1.38  (0.14, 13.4) | 0.783 | 2.11  (0.22, 20.7) | 0.520 | 3.40  (0.35, 33.5) | 0.294 |
| **Renal** | 1.04  (0.67, 1.61) | 0.871 | 0.91  (0.59, 1.40) | 0.680 | **0.46**  **(0.24, 0.90)** | **0.023** | 1.06  (0.68, 1.64) | 0.812 | 0.77  (0.46, 1.30) | 0.334 | **0.37**  **(0.18, 0.75)** | **0.006** | 0.49  (0.22, 1.11) | 0.087 |
| **Haemato-logical** | 0.89  (0.54, 1.45) | 0.629 | 0.84  (0.52, 1.35) | 0.469 | 0.60  (0.29, 1.22) | 0.158 | 1.02  (0.63, 1.66) | 0.941 | 0.77  (0.43, 1.38) | 0.375 | 0.53  (0.25, 1.12) | 0.096 | 0.55  (0.22, 1.35) | 0.194 |
| **BILAG numerical score** | 1.00  (0.98, 1.02) | 0.965 | 1.00  (0.98, 1.02) | 0.971 | **0.92**  **(0.88, 0.96)** | **<0.001** | 0.99  (0.97, 1.02) | 0.668 | 0.98  (0.96, 1.01) | 0.247 | **0.94**  **(0.90, 0.98)** | **0.004** | 0.97  (0.92, 1.01) | 0.115 |
| **Sex**  **Male *** | 1.63  (0.96, 2.78) | 0.071 | 1.61  (0.96, 2.72) | 0.072 | 0.75  (0.34, 1.66) | 0.476 | 1.66  (0.97, 2.82) | 0.063 | 1.51  (0.83, 2.74) | 0.178 | 0.90  (0.42, 1.92) | 0.780 | 1.34  (0.59, 3.06) | 0.482 |
| **Ethnicity****  **Asian**  **White**  **British** | **2.18**  **(1.10, 4.34)**  1.83  (0.95, 3.51) | **0.026**  0.069 | **2.50**  **(1.26, 4.95)**  **2.35**  **(1.23, 4.48)** | **0.009**  **0.010** | 1.15  (0.47, 2.83)  1.55  (0.69, 3.52) | 0.761  0.291 | 1.78  (0.92, 3.46)  1.48  (0.79, 2.77) | 0.089  0.220 | **2.79**  **(1.21, 6.43)**  1.94  (0.86, 4.35) | **0.016**  0.108 | 1.47  (0.54, 3.97)  2.28  (0.92, 5.65) | 0.448  0.075 | 4.32  (0.95, 19.5)  3.78  (0.87, 16.5) | 0.058  0.077 |
| **Diagnosis Age** | 0.96  (0.90, 1.03) | 0.248 | 0.95  (0.90, 1.02) | 0.137 | 0.94  (0.87, 1.02) | 0.148 | **0.94**  **(0.88, 0.99)** | **0.040** | **0.92**  **(0.86, 0.99)** | **0.024** | 0.95  (0.87, 1.03) | 0.190 | 0.93  (0.85, 1.03) | 0.156 |
| **SLICC- SDI score**  **Mild (1)**  **Mod (2)**  **Severe (≥3)** | 0.71  (0.36, 1.41)  0.62  (0.13, 3.06)  0.73  (0.14, 3.67) | 0.328  0.561  0.701 | 1.14  (0.61, 2.13)  0.25  (0.03, 1.99)  1.18  (0.28, 5.02) | 0.677  0.188  0.823 | 0.48  (0.16, 1.38)  3.43e-7  (0.00, Inf)  0.77  (0.09, 6.36) | 0.173  0.985  0.806 | 1.16  (0.61, 2.21)  0.69  (0.14, 3.37)  0.34  (0.04, 2.83) | 0.637  0.644  0.321 | 1.17  (0.57, 2.42)  0.51  (0.06, 4.13)  0.58  (0.07, 4.79) | 0.662  0.526  0.613 | 0.49  (0.17, 1.41)  3.51e-7  (0.00,Inf)  0.78  (0.09, 6.51) | 0.186  0.985  0.822 | 0.59  (0.18, 2.01)  5.82e-7  (0.00, Inf)  1.30  (0.16, 10.9) | 0.404  0.986  0.807 |
| **SLEDAI score** | 0.98  (0.96, 1.01) | 0.272 | 0.98  (0.96, 1.01) | 0.221 | **0.88**  **(0.83, 0.93)** | **<0.001** | 0.98  (0.95, 1.01) | 0.223 | 0.98  (0.95, 1.02) | 0.317 | **0.93**  **(0.88, 0.97)** | **0.001** | **0.94**  **(0.89, 0.99)** | **0.035** |

LLDAS – Lupus low disease activity state. LA - Low Activity. LDA - Low Disease Activity. SLEDAI = systemic lupus erythematosus disease activity index. BILAG = British Isles lupus assessment group. C3 = complement factor 3. C4 = complement factor 4. dsDNA positive = anti-double stranded DNA antibody positivity. Hb = haemoglobin. ESR = erythrocyte sedimentation rate. SLICC-SDI – Systemic Lupus International Collaborating Clinics Standardised Damage Index. *Odds reported as compared to females, females are the reference for this variable. **Odds reported as compared to those of African/Caribbean ethnicity, patients of African/Caribbean ethnicity are the reference for this variable. Variables in bold are significant in these univariable analyses.

**Supplementary Table S4 -** **Two sided t-tests comparing HR for different multivariable PWP-Gap-target models for ‘severe flare’ outcome**

**(original models shown in Table 6 of the main manuscript)**

| **HR’s compared** | **Target 1 achieved** | **Target 2 achieved** | **Adjusted**  **p_c_-value** |
| --- | --- | --- | --- |
| **Low disease activity**  **state definitions** | LLDAS | LA | **<0.001** |
|  | LLDAS | Toronto-LDA | 0.494 |
|  | LA | Toronto-LDA | 0.098 |
| **Remission definitions** | Remission ON-Tx (SLEDAI-defined) | Remission OFF-Tx (SLEDAI-defined) | 0.435 |
| **Low disease activity state vs**  **remission definitions** | LLDAS | Remission ON-Tx (SLEDAI-defined) | 0.306 |
|  | LLDAS | Remission OFF-Tx (SLEDAI-defined) | 1.000 |
|  | LA | Remission ON-Tx (SLEDAI-defined) | **<0.001** |
|  | LA | Remission OFF-Tx (SLEDAI-defined) | **<0.001** |
|  | Toronto-LDA | Remission ON-Tx (SLEDAI-defined) | 1.000 |
|  | Toronto-LDA | Remission OFF-Tx (SLEDAI-defined) | **<0.001** |

LLDAS – Lupus low disease activity state. LA - Low Activity. LDA - Low Disease Activity. SLEDAI = systemic lupus erythematosus disease activity index. P_c_ = Bonferroni adjusted p-value for 10 comparisons.

**Supplementary Table S5 - Two sided t-tests comparing HR for different multivariable PWP-Gap-target models assessing the impact of**

**percentage of cumulative duration in each target on ‘severe flare’ outcome (original models shown in lower section of Table 6 of the main manuscript)**

| **HR’s compared** | **Percentage of cumulative duration**  **in Target 1** | **Percentage of cumulative duration**  **in Target 2** | **Adjusted**  **p_c_-value** |
| --- | --- | --- | --- |
| **Low disease activity**  **state definitions** | Percentage of the cumulative duration in LLDAS | Percentage of the cumulative duration in LA | **0.006** |
|  | Percentage of the cumulative duration in LLDAS | Percentage of the cumulative duration in Toronto-LDA | **0.005** |
|  | Percentage of the cumulative duration in LA | Percentage of the cumulative duration in Toronto-LDA | 0.193 |
| **Remission definitions** | Percentage of the cumulative duration Remission ON-Tx (SLEDAI-defined) | Percentage of the cumulative duration in Remission ON-Tx (BILAG-defined) | **<0.001** |
|  | Percentage of the cumulative duration in Remission ON-Tx (SLEDAI-defined) | Percentage of the cumulative duration in Remission OFF-Tx (SLEDAI-defined) | **0.014** |
|  | Percentage of the cumulative duration in Remission ON-Tx (SLEDAI-defined) | Percentage of the cumulative duration in Remission OFF-Tx (BILAG-defined) | 1.000 |
|  | Percentage of the cumulative duration in Remission ON-Tx (BILAG-defined) | Percentage of the cumulative duration in Remission OFF-Tx (SLEDAI-defined) | **<0.001** |
|  | Percentage of the cumulative duration in Remission ON-Tx (BILAG-defined) | Percentage of the cumulative duration in Remission OFF-Tx (BILAG-defined) | 1.000 |
|  | Percentage of the cumulative duration in Remission OFF-Tx (SLEDAI-defined) | Percentage of the cumulative duration in Remission OFF-Tx (BILAG-defined) | **<0.001** |
| **Low disease activity state vs**  **remission definitions** | Percentage of the cumulative duration in LLDAS | Percentage of the cumulative duration in Remission ON-Tx (SLEDAI-defined) | 0.693 |
|  | Percentage of the cumulative duration in LLDAS | Percentage of the cumulative duration in Remission ON-Tx (BILAG-defined) | 0.387 |
|  | Percentage of the cumulative duration in LLDAS | Percentage of the cumulative duration in Remission OFF-Tx (SLEDAI-defined) | **0.002** |
|  | Percentage of the cumulative duration in LLDAS | Percentage of the cumulative duration in Remission OFF-Tx (BILAG-defined) | 1.000 |
|  | Percentage of the cumulative duration in LA | Percentage of the cumulative duration in Remission ON-Tx (SLEDAI-defined) | 1.000 |
|  | Percentage of the cumulative duration in LA | Percentage of the cumulative duration in Remission ON-Tx (BILAG-defined) | **<0.001** |
|  | Percentage of the cumulative duration in LA | Percentage of the cumulative duration in Remission OFF-Tx (SLEDAI-defined) | 0.102 |
|  | Percentage of the cumulative duration in LA | Percentage of the cumulative duration in Remission OFF-Tx (BILAG-defined) | 1.000 |
|  | Percentage of the cumulative duration in Toronto-LDA | Percentage of the cumulative duration in Remission ON-Tx (SLEDAI-defined) | **0.043** |
|  | Percentage of the cumulative duration in Toronto-LDA | Percentage of the cumulative duration in Remission ON-Tx (BILAG-defined) | **<0.001** |
|  | Percentage of the cumulative duration in Toronto-LDA | Percentage of the cumulative duration in Remission OFF-Tx (SLEDAI-defined) | 1.000 |
|  | Percentage of the cumulative duration in Toronto-LDA | Percentage of the cumulative duration in Remission OFF-Tx (BILAG-defined) | **0.002** |

LLDAS – Lupus low disease activity state. LA - Low Activity. LDA - low disease activity. SLEDAI - systemic lupus erythematosus disease activity index. BILAG - British Isles lupus assessment group. P_c_-value - Bonferroni adjusted p-value for 21 comparisons.

**Supplementary Table S6 - Two sided t-tests comparing different univariable target models based on HR for ‘new damage’ outcome (original models shown in Table 4 of the main manuscript)**

| **HR’s compared** | **Target 1 achieved** | **Target 2 achieved** | **Adjusted**  **p_c_-value** |
| --- | --- | --- | --- |
| **Low disease activity**  **state definitions** | LLDAS | LA | 0.111 |
|  | LLDAS | Toronto-LDA | 1.000 |
|  | LA | Toronto-LDA | 1.000 |
| **Remission definitions** | Remission ON-Tx (SLEDAI-defined) | Remission ON-Tx (BILAG-defined) | 0.991 |
|  | Remission ON-Tx (SLEDAI-defined) | Remission OFF-Tx (SLEDAI-defined) | 1.000 |
|  | Remission ON-Tx (SLEDAI-defined) | Remission OFF-Tx (BILAG-defined) | 0.000 |
|  | Remission ON-Tx (BILAG-defined) | Remission OFF-Tx (SLEDAI-defined) | 1.000 |
|  | Remission ON-Tx (BILAG-defined) | Remission OFF-Tx (BILAG-defined) | **<0.001**** |
|  | Remission OFF-Tx (SLEDAI-defined) | Remission OFF-Tx (BILAG-defined) | **<0.001** |
| **Low disease activity state vs**  **remission definitions** | LLDAS | Remission ON-Tx (SLEDAI-defined) | 1.000 |
|  | LLDAS | Remission ON-Tx (BILAG-defined) | 1.000 |
|  | LLDAS | Remission OFF-Tx (SLEDAI-defined) | 1.000 |
|  | LLDAS | Remission OFF-Tx (BILAG-defined) | **<0.001** |
|  | LA | Remission ON-Tx (SLEDAI-defined) | 0.251 |
|  | LA | Remission ON-Tx (BILAG-defined) | 0.232 |
|  | LA | Remission OFF-Tx (SLEDAI-defined) | 1.000 |
|  | LA | Remission OFF-Tx (BILAG-defined) | **<0.001** |
|  | Toronto-LDA | Remission ON-Tx (SLEDAI-defined) | 1.000 |
|  | Toronto-LDA | Remission ON-Tx (BILAG-defined) | 1.000 |
|  | Toronto-LDA | Remission OFF-Tx (SLEDAI-defined) | 1.000 |
|  | Toronto-LDA | Remission OFF-Tx (BILAG-defined) | **<0.001** |

LLDAS – Lupus low disease activity state. LA - Low Activity. LDA - low disease activity. SLEDAI - systemic lupus erythematosus disease activity index. BILAG - British Isles Lupus Assessment Group. P_c_-value - Bonferroni adjusted p-value for multiple testing (p-value multiplied 21 times).

**Supplementary Box S1 – Describes the Prentice, William and Peterson (PWP-Gap) model and the variables considered within it**

The PWP – Gap model describes an intensity process from the occurrence of an immediately preceding event, with the gap time defined as (t – t_k-1_). The PWP approach is based on a conditional model as an individual is not considered in the risk set for the k^th^ event until experiencing the (k−1)^th^ event. The baseline hazards vary from event to event. The hazard function for the k^th^ event for the i^th^ subject with the Proportional Hazard form is written as:

λ_ik_(t)=λ_0_(t-t_k-1_)exp{β_k_ x_i_(t)}

λ_0k_(t) represents the event-specific baseline hazard for the k^th^ event over time.

Variables considered within the PWP-Gap model:

- Gender (female = reference)
- Ethnicity (Black African or Caribbean as reference, Asian, White British)
- SLICC- SDI Damage index score at study recruitment
- Increasing SLICC SDI score during follow-up
- Target state attainment at any timepoint:
  - LLDAS
  - LA
  - Toronto-LDA
  - Remission ON-Tx (SLEDAI-defined)
  - Remission ON-Tx (BILAG-defined)
  - Remission OFF-Tx (SLEDAI-defined)
  - Remission OFF-Tx (BILAG-defined)
- Percentage of the cumulative duration of follow-up in each target state (as a percentage of the total follow-up period, HR per 1% cumulative duration):
- LLDAS
- LA
- Toronto-LDA
  - Remission ON-Tx (SLEDAI-defined)
  - Remission ON-Tx (BILAG-defined)
  - Remission OFF-Tx (SLEDAI-defined)
  - Remission OFF-Tx (BILAG-defined)

**Supplementary Figure S1 - Venn diagram showing overlap in attainment of different LDA and remission definitions on per visit basis**

**Footnote**

The numbers in the Venn diagram represent individual visits. Of the 4738 total visit: LDA attained at 1368 visits, LLDAS attained at 918 visits,

Toronto LDA attained at 393 visits, Remission on treatment (SLEDAI defined) attainted at 848 visits, Remission on treatment (BILAG defined).

LLDAS – Lupus low disease activity state. LA - Low Activity. LDA – Low Disease Activity. SLEDAI – definition of remission based upon the Systemic Lupus Erythematosus disease activity index. BILAG – definition of remission based upon the British Isles Lupus assessment group score.
